# Intake of dietary flavonoids and risk of epithelial ovarian cancer^1^,^2^,^3^,^4^

**DOI:** 10.3945/ajcn.114.088708

**Published:** 2014-08-20

**Authors:** Aedín Cassidy, Tianyi Huang, Megan S Rice, Eric B Rimm, Shelley S Tworoger

**Affiliations:** 1 From the Department of Nutrition, Norwich Medical School, University of East Anglia, Norwich, United Kingdom (AC); the Department of Epidemiology, Harvard School of Public Health, Boston, MA (TH, MSR, EBR, and SST); and the Channing Division of Network Medicine, Department of Medicine, Brigham and Women's Hospital and Harvard Medical School, Boston, MA (TH, MSR, EBR, and SST).

## Abstract

**Background:** The impact of different dietary flavonoid subclasses on risk of epithelial ovarian cancer is unclear, with limited previous studies that have focused on only a few compounds.

**Objective:** We prospectively examined associations between habitual flavonoid subclass intake and risk of ovarian cancer.

**Design:** We followed 171,940 Nurses’ Health Study and Nurses’ Health Study II participants to examine associations between intakes of total flavonoids and their subclasses (flavanones, flavonols, anthocyanins, flavan-3-ols, flavones, and polymeric flavonoids) and risk of ovarian cancer by using Cox proportional hazards models. Intake was calculated from validated food-frequency questionnaires collected every 4 y.

**Results:** During 16–22 y of follow-up, 723 cases of ovarian cancer were confirmed through medical records. In pooled multivariate-adjusted analyses, total flavonoids were not statistically significantly associated with ovarian cancer risk (HR for the top compared with the bottom quintile: 0.85; 95% CI: 0.66, 1.09; *P*-trend = 0.17). However, participants in the highest quintiles of flavonol and flavanone intakes had modestly lower risk of ovarian cancer than did participants in the lowest quintile, although the *P*-trend was not significant [HRs: 0.76 (95% CI: 0.59, 0.98; *P*-trend = 0.11) and 0.79 (95% CI: 0.63,1.00; *P*-trend = 0.26), respectively]. The association for flavanone intake was stronger for serous invasive and poorly differentiated tumors (comparable HR: 0.68; 95% CI: 0.50, 0.92; *P*-heterogeneity = 0.10, *P*-trend = 0.07) compared with nonserous and less-aggressive tumors. Intakes of other subclasses were not significantly associated with risk. In food-based analyses used to compare subjects who consumed >1 and ≤1 cup black tea/d, the HR was 0.68 (95% CI: 0.51, 0.90; *P* < 0.01).

**Conclusions:** Higher intakes of flavonols and flavanones as well as black tea consumption may be associated with lower risk of ovarian cancer. Additional prospective studies are required to confirm these findings.

See corresponding editorial on page 1217.

## INTRODUCTION

Epithelial ovarian cancer remains a highly lethal malignancy, and to date, few modifiable risk factors have been established (1, 2). With respect to diet, early ecologic studies suggested that a high plant-based diet that is rich in fruit and vegetables may be related to decreased risk, (3–5) although subsequent case-control and prospective studies have yielded inconsistent findings (6–11). Plants contain bioactive constituents called flavonoids that modulate key cellular signaling pathways and regulate multiple cancer-inflammation pathways and epigenetic cofactors. These compounds have the potential to exert chemopreventive effects (12–14), suggesting that flavonoids might be specific components in plants that could reduce ovarian cancer risk.

Flavonoids are present in many foods and beverages including fruit, vegetables, tea, and wine. Flavonoid subclasses commonly consumed in the United States include flavanones, flavonols, anthocyanins, flavan-3-ols, flavones, and polymeric flavonoids. Relatively few studies have examined the association between these compounds and ovarian cancer risk. Case-control studies generally have reported inverse associations between flavonols and flavones and risk of ovarian cancer (15–17). Our previous prospective analysis of 5 specific compounds in these classes also observed suggestive inverse associations (18). However, a subsequent prospective study, which examined associations of flavones and some flavonols across several cancer sites, observed no associations with ovarian cancer (19). Previous studies were limited by relatively small case numbers, and because, until recently, food databases did not contain the comprehensive range of flavonoids present in the diet, no previous studies, to our knowledge, have examined the full range of flavonoid subclasses in relation to ovarian cancer risk. Therefore, we examined the association of 6 flavonoid subclasses, total flavonoid intake, and their main food sources with risk of epithelial ovarian cancer in the Nurses’ Health Study (NHS)^5^ and Nurses’ Health Study II (NHSII), including an examination by tumor subtypes, with more than twice the number of cases than in our previous analysis (18).

## SUBJECTS AND METHODS

### Study population

The NHS commenced in 1976 when 121,700 US women aged 30–55 y completed a mailed questionnaire about known and suspected risk factors for cancer and cardiovascular disease. In 1989, a younger cohort of 116,430 women aged 25–42 y were enrolled in the NHSII by using similar questionnaires. Participants have completed follow-up questionnaires biennially, providing updated information on lifestyle factors and disease diagnoses. Every 2–4 y, women receive semiquantitative food-frequency questionnaires (FFQs) (20, 21). To maximize the statistical power, we included participants who had answered at least one FFQ since 1984 for NHS participants or 1991 for NHSII participants. As a result, individuals entered the analysis at the questionnaire cycle in which they completed their first FFQ. Participants who reported a bilateral oophorectomy, menopause as result of pelvic irradiation, or cancer other than nonmelanoma skin cancer before study entry, did not complete an FFQ during follow-up, or had implausible values for total caloric intake (<500 or >3500 kcal/d) were excluded, which resulted in the inclusion of 82,289 women from the NHS and 89,651 women from the NHSII for a total of 171,940 participants. The Institutional Review Board at Brigham and Women's Hospital reviewed and approved this study, and participants provided implied consent by returning their questionnaires.

### Outcome assessment

The outcome was incident epithelial ovarian cancer. We collected information about new ovarian cancer diagnoses on each biennial questionnaire. For 90% of reported cases as well as deaths that were ovarian cancer identified through family members, the US National Death Index, or the US Postal Service, we obtained medical records related to the diagnosis. When we were unable to obtain medical records, we linked to the appropriate cancer registry to confirm the diagnosis. An estimated 98% of all deaths in the NHS and NHSII are captured through the National Death Index (22, 23). A gynecologic pathologist blinded to exposure status reviewed medical records to confirm the diagnosis and abstract stage, histology, and invasiveness.

### Dietary assessment

Dietary intake data were assessed in 1984, 1986, and every 4 y thereafter in the NHS. Similar FFQs were initially administered in 1991 and subsequently every 4 y in the NHSII. A database for the assessment of intakes of different flavonoid subclasses was constructed as previously described (24) by using USDA databases for the flavonoid and proanthocyanin contents of foods as primary data sources (25, 26).

Briefly, intakes of individual compounds were calculated as the sum of the consumption frequency of each food multiplied by the content of the specific flavonoid for the specified portion size. We derived intakes of the subclasses commonly consumed in the US diet, specifically flavanones (eriodictyol, hesperetin, and naringenin), anthocyanins (cyanidin, delphinidin, malvidin, pelargonidin, petunidin, and peonidin), flavan-3-ols (catechins and epicatachin), flavonols (quercetin, kaempferol, myricetin, and isohamnetin), flavones (luteolin and apigenin), flavonoid polymers (proanthocyanidins, theaflavins, and thearubigins), and proanthocyanidins alone. Cumulative average intakes (energy adjusted) were calculated for a given questionnaire cycle by averaging the intake for current and preceding FFQs to assess long-term flavonoid intake and minimize the within-person variation; for questionnaire cycles without an FFQ, we used the cumulative average exposure assessed through the previous cycle. The validity and reproducibility of the FFQs have been reported previously, and correlations between major dietary sources of flavonoids, including fruit, vegetables, tea, and wine, measured by diet records and the FFQs were 0.70, 0.50, 0.77, and 0.83, respectively (27–29). We also conducted food-based analyses of the following main sources of flavonols, proanthocyanidins, flavanones, and flavones: tea, onions, celery, apples, and citrus fruit and juices.

### Statistical analysis

Participants contributed person-time of follow-up from the date of return of the first available FFQ to the date of any cancer diagnosis, bilateral oophorectomy, menopause as a result of pelvic irradiation, death, or end of follow-up (June 2010 for the NHS and June 2011 for the NHSII). We used Cox proportional hazard regression for time-varying covariates to estimate the HR and 95% CI for flavonoid intake in relation to risk of ovarian cancer with the lowest quintile of intake as the referent group. We used a 4-y lag between exposure assessment and disease incidence to address the issue that early symptoms of undiagnosed ovarian cancer may alter habitual food intake. For example, we used cumulative average intake from 1984 to 1990 as the exposure for the follow-up period from 1994 to 1998 and cumulative average intake from 1984 to 1994 for follow-up from 1998 to 2002. Because of relatively low within-individual correlations over time in flavonoid intake, if a woman did not answer a particular FFQ, she did not contribute person-time until she answered a subsequent FFQ.

Analyses were conducted within each cohort separately and pooled (stratifying by cohort) because no heterogeneity in associations was observed by using a random-effects meta-analysis (30). To facilitate the comparison of results, common dietary quintiles derived from the pooled distribution of both studies were used. All analyses were stratified by age in months and the calendar year of the current questionnaire cycle, and we further controlled for menopausal status (premenopausal compared with postmenopausal), the duration of oral contraceptive use (never and <1, 1–5, and >5 y), type (estrogen only, estrogen plus progesterone, and other) and duration of postmenopausal hormone (never and <5, 5–10 and >10 y), parity (nulliparous and 1, 2, 3, and >3 children), history of tubal ligation (yes or no), history of hysterectomy (yes or no), family history of breast or ovarian cancer (yes or no), cumulative updated average total energy intake (kcal/d, in quintiles), caffeine intake (mg/d, in quintiles), and lactose intake (g/d, in quintiles). Flavonoid intakes were adjusted for total energy intake by using the residual method (21).

In addition, we examined associations by histologic subtypes (serous invasive and poorly differentiated cancers compared with nonserous) and tumor aggressiveness [defined by rapidly fatal (death ≤3 y of an ovarian cancer diagnosis) compared with less aggressive (all others)] (31) by using competing risks Cox models because there has been evidence that the cause of ovarian cancer may differ by histologic subtype and aggressiveness (32). For the tumor-aggressiveness analysis, the end of follow-up was June 2008 for the NHS and June 2009 for the NHSII to ensure ≥3 y of follow-up after diagnosis (mortality follow-up was updated through to 2012 for both cohorts). We also evaluated whether the association between flavonoid intake and incidence of ovarian cancer differed by menopausal status at diagnosis. All analyses were conducted with SAS software (version 9; SAS Institute Inc). All *P* values were 2 sided.

## RESULTS

During 1,139,897 person-years of follow-up in the NHS and 1,128,526 person-years of follow-up in the NHSII, 723 cases of incident ovarian cancer were confirmed. At the midpoint of follow-up, women with higher compared with lower total flavonoid intakes were slightly older and had lower total energy and lactose intakes (**Table 1**). The flavonoid polymer subclass contributed most to total flavonoid intake (IQR: 95.4–274.7 mg/d), whereas IQRs for flavonol and flavanone intakes ranged from 10.2 to 20.9 and 15.8 to 52.6 mg/d, respectively.

**TABLE 1:** Age-standardized characteristics of the study population at follow-up midpoint by quintiles of total flavonoid intake in the NHS (1998) and the NHSII (2001)*^1^*

	NHS			NHSII		
	Q1	Q3	Q5	Q1	Q3	Q5
Subjects (*n*)	10,035	10,024	10,027	11,877	11,901	11,887
Age (y)	63.3 ± 7.1*^2^*	64.4 ± 7.0	64.6 ± 7.3	46.1 ± 4.6	46.5 ± 4.6	47.1 ± 4.5
History of tubal ligation (%)	22	21	20	26	22	23
History of hysterectomy (%)	22	23	22	9	9	9
Family history of breast cancer or ovarian cancer (%)	16	17	16	13	13	14
Parous (%)	95	95	95	83	82	81
No. of children in parous women	3.3 ± 1.6	3.2 ± 1.5	3.2 ± 1.5	2.3 ± 0.9	2.3 ± 1.0	2.3 ± 0.9
Oral contraceptive use (%)	49	49	48	86	85	85
Duration of oral contraceptive use (mo)*^3^*	52.5 ± 47.7	50.9 ± 46.7	49.7 ± 45.6	69.8 ± 60.1	66.9 ± 59.1	68.0 ± 60.8
Postmenopausal (%)	93	93	93	15	15	16
Estrogen-only PMH use (%)*^4^*	19	21	18	4	4	4
Duration of estrogen-only PMH use ( mo)*^3^*	90.0 ± 77.8	91.3 ± 80.8	87.5 ± 78.0	1.7 ± 2.0	2.6 ± 3.1	2.1 ± 1.8
Estrogen-progestin PMH use (%)*^4^*	31	34	31	53	54	55
Duration of estrogen-progestin PMH use (mo)*^3^*	58.0 ± 43.5	59.8 ± 43.3	60.5 ± 43.0	3.0 ± 2.6	2.8 ± 2.3	2.7 ± 2.3
Total energy intake (cal/d)	1658 ± 526	1763 ± 523	1679 ± 518	1748 ± 550	1854 ± 534	1765 ± 534
Caffeine (mg/d)*^5^*	237 ± 236	190 ± 195	224 ± 185	244 ± 234	213 ± 204	245 ± 193
Lactose (g/d)*^5^*	16.6 ± 13.8	16.5 ± 11.9	15.6 ± 11.6	17.7 ± 14.9	18.3 ± 13.0	16.9 ± 12.7
Flavonol (mg/d)*^5^*	10.2 ± 6.2	15.1 ± 6.8	29.6 ± 13.3	9.7 ± 5.9	14.8 ± 6.9	30.6 ± 14.0
Flavone (mg/d)*^5^*	1.2 ± 0.8	2.5 ± 1.2	2.8 ± 1.9	1.0 ± 0.6	2.1 ± 1.1	2.4 ± 1.7
Flavanone (mg/d)*^5^*	23.7 ± 21.8	55.6 ± 36.4	62.3 ± 54.1	16.2 ± 15.9	41.2 ± 32.7	46.6 ± 49.1
Flavan-3-ol (mg/d)*^5^*	9.3 ± 5.0	19.9 ± 8.3	117 ± 87	9.1 ± 4.9	20.3 ± 8.3	129 ± 95
Polymers (mg/d)*^5^*	55.6 ± 22.4	126 ± 32	465 ± 278	59.9 ± 20.7	135 ± 29	511 ± 305
Anthocyanidin (mg/d)*^5^*	5.8 ± 5.0	14.7 ± 9.3	23.0 ± 27.0	5.7 ± 4.7	15.1 ± 9.4	24.8 ± 35.0
Proanthocyanidin (mg/d)*^5^*	54.6 ± 22.9	114 ± 37	191 ± 87	57.9 ± 21.7	119 ± 37	199 ± 93
Flavan-3-ols and polymers (mg/d)*^5^*	58.1 ± 23.1	122 ± 36	264 ± 112	60.6 ± 21.6	128 ± 35	280 ± 122
Total flavonoids (mg/d)*^5^*	107 ± 30	237 ± 19	699 ± 353	102 ± 28	230 ± 20	743 ± 397

1 NHS, Nurses’ Health Study; NHSII, Nurses’ Health Study II; PMH, postmenopausal hormone; Q, quintile.

2 Mean ± SD (all such values).

3 In ever users.

4 In postmenopausal women.

5 Intake adjusted for total energy by using the nutrient residual method.

In pooled multivariate-adjusted analyses, total flavonoids were not significantly associated with ovarian cancer risk (HR for top compared with bottom quintiles: 0.85; 95% CI: 0.66, 1.09; *P*-trend = 0.17) (**Table 2**). However, participants in the highest quintiles of flavonol and flavanone intakes had modestly lower risk of ovarian cancer than that of participants in the lowest quintiles [HRs: 0.76 (95% CI: 0.59, 0.98; *P*-trend = 0.11) and 0.79 (95% CI: 0.63, 1.00; *P*-trend = 0.26), respectively]. Intakes of other subclasses were not significantly associated with ovarian cancer risk. We also examined the contribution of individual constituents of flavonol and flavanone subclasses (*see* Supplemental Table 1 under “Supplemental data” in the online issue), but none reached statistical significance. In stratified analysis, we did not observe an interaction with menopausal status [HR for top compared with bottom quintiles for flavonols: 0.71 (95% CI: 0.41, 1.20) in premenopausal women; 0.77 (95% CI: 0.58, 1.02) in postmenopausal women; *P*-interaction = 0.59; HRs for top compared with bottom quintiles for flavanones: 0.73 (95% CI: 0.43, 1.26) in premenopausal women; and 0.79 (95% CI: 0.60, 1.03) in postmenopausal women; *P*-interaction = 0.33]. We generally did not observe heterogeneity by cohort (data not shown).

**TABLE 2:** Cohort-specific and pooled HRs (95% CIs) for the association between total flavonoid and subclass intakes and incident epithelial ovarian cancer in the NHS and the NHSII*^1^*

	Quintile of intakes^2^					
	1	2	3	4	5	*P-*trend
Total flavonoids (mg/d)	117.1*^3^*	186.7	258.0	373.1	713.4	—
NHS						
No. of cases/person-years	112/228,949	113/228,485	135/228,938	109/231,309	93/222,217	—
HR (95% CI)	1.00 (reference)	0.91 (0.70, 1.19)	1.06 (0.82, 1.36)	0.86 (0.66, 1.13)	0.83 (0.63, 1.10)	0.14
NHSII						
No. of cases/person-years	26/224,873	30/225,249	29/224,471	47/221,993	29/231,940	—
HR (95% CI)	1.00 (reference)	1.11 (0.65, 1.89)	1.04 (0.61, 1.77)	1.72 (1.05, 2.79)	0.93 (0.54, 1.60)	0.80
Pooled HR (95% CI)	1.00 (reference)	0.96 (0.76, 1.21)	1.07 (0.85, 1.34)	1.03 (0.81, 1.30)	0.85 (0.66, 1.09)	0.17
Flavonols (mg/d)	7.4	11.0	14.4	19.2	30.2	—
NHS						
No. of cases/person-years	130/261,685	125/243,249	111/228,293	117/214,986	79/191,684	—
HR (95% CI)	1.00 (reference)	0.92 (0.71, 1.18)	0.84 (0.65, 1.09)	0.91 (0.70, 1.19)	0.73 (0.55, 0.98)	0.05
NHSII						
No. of cases/person-years	24/191,652	17/210,016	35/225,477	51/238,637	34/262,743	—
HR (95% CI)	1.00 (reference)	0.61 (0.33, 1.14)	1.13 (0.67, 1.91)	1.49 (0.91, 2.45)	0.86 (0.50, 1.47)	0.90
Pooled HR (95% CI)	1.00 (reference)	0.87 (0.69, 1.09)	0.90 (0.71, 1.14)	1.03 (0.82, 1.30)	0.76 (0.59, 0.98)	0.11
Flavones (mg/d)	0.7	1.2	1.6	2.2	3.2	—
NHS						
No. of cases/person-years	77/174,817	103/195,302	109/224,593	137/260,856	136/284,329	—
HR (95% CI)	1.00 (reference)	1.08 (0.80, 1.45)	1.01 (0.75, 1.35)	1.04 (0.78, 1.38)	0.89 (0.67, 1.19)	0.24
NHSII						
No. of cases/person-years	44/280,972	29/259,602	33/229,222	27/192,602	28/166,128	—
HR (95% CI)	1.00 (reference)	0.68 (0.42, 1.09)	0.81 (0.51, 1.28)	0.82 (0.50, 1.33)	0.90 (0.55, 1.45)	0.93
Pooled HR (95% CI)	1.00 (reference)	0.95 (0.74, 1.22)	0.94 (0.74, 1.21)	0.97 (0.76, 1.23)	0.87 (0.68, 1.11)	0.31
Flavanones (mg/d)	7.8	18.6	31.2	47.5	75.8	—
NHS						
No. of cases/person-years	97/181,515	82/187,558	107/224,788	139/266,853	137/279,184	—
HR (95% CI)	1.00 (reference)	0.75 (0.56, 1.01)	0.82 (0.62, 1.08)	0.88 (0.68, 1.15)	0.75 (0.57, 0.97)	0.16
NHSII						
No. of cases/person-years	40/273,465	33/267,747	32/229,213	31/185,730	25/172,370	—
HR (95% CI)	1.00 (reference)	0.86 (0.54, 1.36)	0.93 (0.58, 1.50)	1.11 (0.69, 1.79)	0.93 (0.56, 1.55)	0.86
Pooled HR (95% CI)	1.00 (reference)	0.78 (0.61, 1.01)	0.85 (0.67, 1.08)	0.93 (0.74, 1.17)	0.79 (0.63, 1.00)	0.26
Flavan-3-ols (mg/d)	9.3	16.9	28.2	54.2	133.7	—
NHS						
No. of cases/person-years	111/234,555	122/223,801	122/228,573	114/231,532	93/221,436	—
HR (95% CI)	1.00 (reference)	1.07 (0.83, 1.39)	1.03 (0.80, 1.34)	0.99 (0.76, 1.30)	0.90 (0.68, 1.19)	0.25
NHSII						
No. of cases/person-years	25/218,908	26/229,718	46/224,960	35/222,082	29/232,857	—
HR (95% CI)	1.00 (reference)	0.97 (0.56, 1.69)	1.63 (0.99, 2.67)	1.29 (0.76, 2.17)	0.95 (0.55, 1.65)	0.43
Pooled HR (95% CI)	1.00 (reference)	1.06 (0.84, 1.34)	1.16 (0.92, 1.45)	1.06 (0.83, 1.34)	0.91 (0.71, 1.16)	0.16
Polymers (mg/d)	61.4	106.2	156.3	240.7	496.5	—
NHS						
No. of cases/person-years	122/255,573	130/227,280	116/218,260	105/222,406	89/216,378	—
HR (95% CI)	1.00 (reference)	1.08 (0.84, 1.39)	0.99 (0.76, 1.28)	0.91 (0.70, 1.18)	0.83 (0.63, 1.10)	0.08
NHSII						
No. of cases/person-years	22/197,300	30/226,489	37/235,387	43/231,374	29/237,976	—
HR (95% CI)	1.00 (reference)	1.20 (0.68, 2.08)	1.34 (0.78, 2.29)	1.60 (0.95, 2.69)	0.97 (0.55, 1.72)	0.57
Pooled HR (95% CI)	1.00 (reference)	1.10 (0.88, 1.38)	1.05 (0.83, 1.33)	1.03 (0.82, 1.31)	0.85 (0.66, 1.09)	0.07
Anthocyanins (mg/d)	2.5	5.2	8.9	14.0	23.9	—
NHS						
No. of cases/person-years	99/210,408	96/227,476	127/245,131	138/240,150	102/216,733	—
HR (95% CI)	1.00 (reference)	0.82 (0.62, 1.09)	1.00 (0.77, 1.31)	1.11 (0.85, 1.44)	0.85 (0.64, 1.13)	0.73
NHSII						
No. of cases/person-years	33/244,670	34/226,342	25/208,143	23/213,040	46/236,331	—
HR (95% CI)	1.00 (reference)	1.07 (0.66, 1.73)	0.79 (0.46, 1.34)	0.72 (0.42, 1.24)	1.22 (0.77, 1.93)	0.40
Pooled HR (95% CI)	1.00 (reference)	0.87 (0.68, 1.12)	0.96 (0.76, 1.22)	1.03 (0.82, 1.31)	0.95 (0.75, 1.21)	0.85
Proanthocyanidins (mg/d)	54.0	83.0	108.6	139.4	196.8	—
NHS						
No. of cases/person-years	126/253,021	117/236,742	110/229,082	122/218,161	87/202,890	—
HR (95% CI)	1.00 (reference)	0.94 (0.73, 1.22)	0.88 (0.68, 1.15)	1.00 (0.77, 1.29)	0.76 (0.58, 1.01)	0.10
NHSII						
No. of cases/person-years	24/200,236	23/216,890	36/224,990	29/235,608	49/250,801	—
HR (95% CI)	1.00 (reference)	0.87 (0.49, 1.54)	1.25 (0.74, 2.11)	0.95 (0.55, 1.64)	1.45 (0.88, 2.38)	0.07
Pooled HR (95% CI)	1.00 (reference)	0.93 (0.74, 1.17)	0.95 (0.76, 1.20)	0.98 (0.78, 1.24)	0.92 (0.73, 1.16)	0.64

1 Stratified by age and calendar time and adjusted for menopausal status, duration of oral contraceptive use, parity, history of tubal ligation, history of hysterectomy, duration of postmenopausal hormone use by type, family history of breast or ovarian cancer, quintiles of cumulative updated energy-adjusted lactose intake and caffeine intake, and quintiles of cumulative updated total energy intake. Further stratified by cohort in the pooled analysis. Median values in each quintile were used to test for a linear trend. Cox proportional hazards models were used for all analyses. NHS, Nurses’ Health Study; NHSII, Nurses’ Health Study II.

2 Quintiles were based on the distribution from the pooled study population.

3 Median (all such values).

We examined associations by histologic subtype by comparing serous and poorly differentiated tumors (*n* = 442) with nonserous tumors (*n* = 274). There was a suggestion that flavanones were more-strongly associated with lower risk of serous invasive and poorly differentiated tumors (HR for top compared with bottom quintiles: 0.68; 95% CI: 0.50, 0.92) than risk of nonserous tumors (HR for top compared with bottom quintiles: 1.04; 95% CI: 0.72, 1.50; *P*-heterogeneity = 0.10; **Table 3**). Apart from flavones (data not shown), no heterogeneity in associations by subtype was observed for other flavonoid subclasses (*P*-heterogeneity > 0.52). In general, we did not observe different associations by tumor aggressiveness, although higher proanthocyanidin intake was associated with suggestively lower risk of rapidly fatal tumors, but not less-aggressive tumors (HR for top compared with bottom quintiles: 0.70; 95% CI: 0.48, 1.04), than risk of less-aggressive tumors (HR for top compared with bottom quintile: 1.06; 95% CI: 0.78, 1.45; *P*-heterogeneity = 0.04).

**TABLE 3:** HRs (95% CIs) for the association between intakes of total flavonoids, flavonols, and flavanones and incident epithelial ovarian cancer by histologic subtype and tumor aggressiveness in the NHS and NHSII*^1^*

	Histologic subtypes (*n* = 723)			Tumor aggressiveness (*n* = 668)		
	Serous and poorly differentiated (*n* = 446)	Nonserous (*n* = 277)	*P*-heterogeneity	Rapidly fatal (*n* = 261)	Less aggressive (*n* = 407)	*P*-heterogeneity
Total flavonoids						0.49
HR (95% CI)	0.82 (0.60, 1.13)	0.86 (0.58, 1.27)	—	0.71 (0.47, 1.06)	0.88 (0.63, 1.22)
* P-*trend	0.22	0.32	0.98	0.09	0.28
Flavonols						0.78
HR (95% CI)	0.75 (0.54, 1.03)	0.70 (0.47, 1.03)	—	0.70 (0.47, 1.05)	0.74 (0.53, 1.04)
* P-*trend	0.15	0.20	0.92	0.19	0.21
Flavanones						0.94
HR (95% CI)	0.68 (0.50, 0.92)	1.04 (0.72, 1.50)	—	0.83 (0.57, 1.22)	0.77 (0.56, 1.05)
* P-*trend	0.07	0.53	0.10	0.49	0.33

1 HRs for comparison of highest with lowest quintiles stratified by age, calendar time, and cohort and adjusted for the duration of oral contraceptive use, parity, history of tubal ligation, history of hysterectomy, duration of postmenopausal hormone use by type, family history of breast or ovarian cancer, and quintiles of energy-adjusted lactose, caffeine, and total energy intakes. Tumor aggressiveness was defined as rapidly fatal within 3 y of ovarian cancer diagnosis. To allow ≥3 y postdiagnostic follow-up, the endpoint for rapidly fatal ovarian cancer was June 2008 for the NHS and June 2009 for the NHSII, respectively. Cox proportional hazards models were used to estimate HRs, and median values in each quintile were used to test for a linear trend. A random-effects meta-analysis method was used for the test of heterogeneity. NHS, Nurses’ Health Study; NHSII, Nurses’ Health Study II.

We conducted food-based analyses for main dietary sources of flavonols and flavonols. Main dietary sources of flavonols were black tea (31%), onions (20%), and apples (10%), whereas citrus fruit (36%; 27% from orange intake) and juices (63%; 54% from orange juice) were the main dietary sources for flavanones (**Table 4**). To examine associations with the main dietary source of flavonols, we compared subjects who consumed >1 cup black tea/d with those who rarely or never drank tea and observed significantly lower risk of ovarian cancer (HR: 0.69; 95% CI: 0.52, 0.93; *P*-trend = 0.03) (Table 4). We also compared subjects who drank >1 cup black tea/d with those who drank ≤1 cup/d and observed similar inverse associations (HR: 0.68; 95% CI: 0.51, 0.90; *P* < 0.01). For other main foods that contributed to intake (apple; grapefruit and grapefruit juice; onion; and orange and orange juice), we did not observe significant associations.

**TABLE 4:** Pooled HRs (95% CIs) for the association between intakes of flavonoid-rich food and incident epithelial ovarian cancer in the NHS and NHSII*^1^*

			Values			*P-*trend
Frequency of servings	<1/wk	1/wk	2–6/wk	1/d	>1/d	
Tea						
No. of cases	395	65	103	90	54	—
HR (95% CI)	1.00 (reference)	1.07 (0.82, 1.40)	1.02 (0.82, 1.28)	1.11 (0.87, 1.40)	0.69 (0.52, 0.93)	0.03
Frequency of servings	<1/mo	1–3/mo	1/wk	2–4/wk	≥5/wk	—
Apple						
No. of cases	96	181	136	198	97	—
HR (95% CI)	1.00 (reference)	0.93 (0.73, 1.20)	0.87 (0.67, 1.14)	0.99 (0.77, 1.27)	0.99 (0.74, 1.33)	0.52
Celery						
No. of cases	163	210	167	135	43	—
HR (95% CI)	1.00 (reference)	0.96 (0.78, 1.18)	0.99 (0.79, 1.23)	1.02 (0.80, 1.29)	0.75 (0.53, 1.06)	0.21
Grapefruit/grapefruit juice*^2^*						
No. of cases	315	142	132	75	49	—
HR (95% CI)	1.00 (reference)	0.97 (0.80, 1.19)	1.09 (0.88, 1.35)	0.87 (0.67, 1.13)	0.95 (0.70, 1.30)	0.50
Onion						
No. of cases	303	193	95	74	39	—
HR (95% CI)	1.00 (reference)	1.09 (0.91, 1.31)	0.93 (0.73, 1.17)	1.05 (0.81, 1.35)	0.99 (0.71, 1.40)	0.91
Orange						
No. of cases	189	202	152	125	44	—
HR (95% CI)	1.00 (reference)	0.93 (0.76, 1.13)	1.11 (0.89, 1.37)	1.00 (0.79, 1.26)	0.88 (0.63, 1.23)	0.61
Frequency of servings	<1/mo	1–3/mo	1/wk	2–6/wk	≥1/d	—
Orange juice						
No. of cases	193	121	79	161	157	—
HR (95% CI)	1.00 (reference)	0.94 (0.75, 1.19)	0.82 (0.63, 1.07)	0.86 (0.70, 1.07)	0.83 (0.67, 1.04)	0.16

1 Stratified by age, calendar time, and cohort and adjusted for menopausal status, duration of oral contraceptive use, parity, history of tubal ligation, history of hysterectomy, duration of postmenopausal hormone use by type, family history of breast or ovarian cancer, quintiles of cumulative updated energy-adjusted lactose and caffeine intake, and quintiles of cumulative updated total energy intake. Number of cases were slightly different by food because of missing values. Median values in each category were used to test for a linear trend. Cox proportional hazards models were used for all analyses. NHS, Nurses’ Health Study; NHSII, Nurses’ Health Study II.

2 Since 2002 in the NHS and 1999 in the NHSII, grapefruit and grapefruit juice were asked in one question.

## DISCUSSION

In this large prospective cohort study we observed that participants who had the highest habitual intakes of flavonols and flavanones had significantly lower risk of epithelial ovarian cancer. Associations for flavanones were more strongly associated with lower risk of serous and poorly differentiated tumors, a more-aggressive disease phenotype. Main sources of these compounds include tea and citrus fruits or juice, foods and drinks that are readily incorporated into the habitual diet, suggesting that simple changes in food intake could have an impact on ovarian cancer risk. To our knowledge, this is the only prospective study to comprehensively examine the 6 major flavonoid subclasses present in the diet with ovarian cancer risk and the first to investigate the impact of polymers and anthocyanins.

In the current study, we observed significantly lower risk of high intakes of flavonols and flavanones only for women in the top quintile (compared with the bottom quintile) of intakes. This results may have been because the absolute range in intake across the 3 middle quintiles compared with the lowest quintile was relatively small (eg, 10 mg for flavonols and 33 mg for flavanones); the measurement error in these middle quintiles may have obscured an association. Another possibility is that there was a threshold effect. Previous data suggested that there may be a threshold dose required for biological effects and low intakes are unlikely to be bioactive (33). When we compared extreme deciles of intake, subjects in the top decile had 29% (flavonols) and 28% (flavanones) reductions in risk of ovarian cancer, which suggested a continual dose-response at higher habitual intakes.

Results of this study were consistent with our earlier study that evaluated several individual compounds present in the 2 flavonoid subclasses flavonols and flavones (18, 19). In our previous prospective study (*n* = 346 cases), we examined the only 5 flavonoid constituents available in food-composition tables at the time [3 flavonols (myricetin, kaempferol, and quercetin) and the flavones (apigenin, luteolin)]. We observed significant inverse trends for all 3 flavonols and luteolin, although after multivariate adjustment, only kaempferol and luteolin remained associated with risk (18). In the Women's Health Study, no associations were observed for these flavonoids and ovarian cancer risk (19), although the number of cases was small (*n* = 141). However, this study did observe a small increase in risk of endometrial and breast cancer when multivariable HRs between highest and lowest quintiles of intake were compared (HRs: 1.15 and 1.03 for endometrial and breast cancer, respectively) for total flavonoid intake (19). In a recent prospective study that integrated a wide range of flavonoid subclasses, no associations between individual flavonoid subclasses and breast cancer risk were observed (34).

Potential anticancer effects and molecular mechanism of flavonols on ovarian cancer are not well understood, but a number of flavonols, including quercetin, myricetin, and kaempferol, are potential modulators of cellular signaling pathways providing plausible mechanisms by which flavonols could reduce cancer risk (35). Specifically, in vitro data have suggested that quercetin can inhibit the proliferation of ovarian cancer cell lines with inhibitory effects observed at exposures observed in human diets (5 μg/mL). The mechanism appears to be through the activation of autophagy, induction of apoptosis, and inhibition of angiogenesis (36, 37). These laboratory data have been supported by a recent animal study in which the intravenous administration of high doses of quercetin (60 mg/kg) to athymic nude mice with xenograft ovarian tumors inhibited angiogenesis and apoptosis in vivo (36). Furthermore, kaempferol and the flavones (apigenin and luteolin) appeared to inhibit cell growth at the 5-μmol/L amount in part via actions on vascular endothelial growth factor (38). Kaempferol at supraphysiologic doses also may exert a chemopreventive action through the blocking of phosphoinositide 3-kinase/protein kinase B signaling pathways and the subsequent suppression of activator protein-1 and nuclear transcription factor κB, which are important transcription factors for neoplastic transformation (35).

For flavanones, with the comparison of extreme quintiles of intake, we observed 21% lower risk of ovarian cancer with higher flavonone intake (median intake: 75.8 mg/d). To our knowledge, this is the first population-based study to suggest a chemopreventive effect for flavanones on ovarian cancer risk. Although potential anticancer effects for several flavanones, including hesperetin (and its glycoside hesperidin) and naringenin, have been described, including effects on inhibiting cell growth via cell cycle arrest and p53-dependent apoptosis (39), the focus to date has been on other cancer sites than the ovary. Although some biological data support that certain flavan-3-ols can increase apoptosis in ovarian cancer cell lines (40), we did not observe an association with this subclass of flavonoids. Earlier versions of our FFQs in the NHS did not capture all foods that may be important sources of this specific flavonoid subclass, such as dark chocolate, which potentially introduced a measurement error.

Available literature from both a molecular and epidemiologic perspective has provided some evidence for a potential role for tea in ovarian cancer prevention (40–42). In our confirmatory food-based analysis of top sources of flavonoid subclasses, we showed significantly lower risk of ovarian cancer with increased intake of black tea. These data are in agreement with our earlier prospective study (18) and support the observed beneficial effects of black tea on ovarian cancer risk from other previous prospective studies (18, 43–46). In a recent meta-analysis (42), black tea consumption was inversely associated with risk (combined HR: 0.73; 95% CI: 0.57, 0.93) in the prospective cohort studies.

Subtypes of ovarian carcinomas have different molecular, pathologic, and clinical characteristics, which support the notion that this histologic diversity may constitute several distinctive entities rather than one single disease (47–49), and evidence has suggested that some ovarian cancer risk-factor associations can differ by histologic subtype (49). We observed a suggestively stronger inverse association between higher flavanone intake and risk of serous tumors. However, there was no association with nonserous tumors. Because serous tumors tend to have poorer outcomes, this outcome may have important implications for prevention. However, we did not observe differences in the association for rapidly fatal compared with less-aggressive tumors.

Strengths of our study included its prospective design, large sample size with long-term follow-up, histologic subtype characterization, repeated measures of dietary intake, comprehensive assessment of the range of flavonoid subclasses present in the habitual diet, and detailed data on ovarian cancer risk factors. Our study also had some limitations. Although we adjusted for possible confounders that are strongly associated with risk of ovarian cancer, there was still the possibility of residual confounding from unmeasured factors. However, because of our detailed and updated adjustment for potential confounders, it was unlikely that these would have accounted fully for the observed results. Also, mean cumulative dietary flavonoid intakes were calculated from a database developed from recent USDA databases with additional input from other sources (24); however, flavonoid contents of foods can vary depending on growth and processing conditions. Despite this variation, these data allowed us to rank-order intakes and compare high with low intakes in large population groups. Correlations between the major dietary sources of flavonoids (tea, fruit, vegetables, and wine) and flavonoid intake have been determined for our FFQ (27, 28), and in a recent flavonoid biomarker study, similar correlations between the sum of 7 flavonoid biomarkers measured in 24-h urine samples were observed (0.43–0.66) (50).

In conclusion, our data suggest that specific bioactive compounds, including flavonols and flavanones, present in plant-based foods may be associated with lower risk of ovarian cancer. In particular, black tea intake was associated with lower risk, which is already commonly consumed in many countries. Additional prospective studies and mechanistic studies are required to confirm these findings and determine whether the effect of diet varies with different subtypes of ovarian cancer because of their different molecular, pathological, and clinical characteristics.

## Supplementary Material

Supplemental data

## Footnotes

5 Abbreviations used: FFQ, food-frequency questionnaire; NHS, Nurses’ Health Study; NHSII, Nurses’ Health Study II.
