# Supplementary material for: Intake of dietary flavonoids and risk of epithelial ovarian cancer1
Source: Am J Clin Nutr. 2014 Aug 20;100(5):1344–51. doi: 10.3945/ajcn.114.088708 (PMC4196485; doi:10.3945/ajcn.114.088708)
Supplement: Supplemental data [file supp_100_5_1344__index.html]

Intake of dietary flavonoids and risk of epithelial ovarian cancer — Intake of dietary flavonoids and risk of epithelial ovarian cancer — Supplemental data 

# Intake of dietary flavonoids and risk of epithelial ovarian cancer

## Supplemental data

**Files in this Data Supplement:**

- Supplemental data - Table 1
