# Supplementary material for: Intake of dietary flavonoids and risk of epithelial ovarian cancer1
Source: Am J Clin Nutr. 2014 Aug 20;100(5):1344–51. doi: 10.3945/ajcn.114.088708 (PMC4196485; doi:10.3945/ajcn.114.088708)
Supplement: Supplemental data [file 114.088708_ajcn088708SupplementaryData1.docx]

| Supplemental Table 1. Pooled hazard ratio (HR) and 95% confidence interval (CI) for the association between individual constituents of the flavonol and flavanones sub-class and incident epithelial ovarian cancer in the Nurses' Health Study (NHS) and the Nurses' Health Study II (NHSII)^1^ | | | | | | |
| --- | --- | --- | --- | --- | --- | --- |
|  |  |  |  |  |  |  |
|  |  |  |  |  |  |  |
|  | Quintiles of intakes^2^ | | | | |  |
|  | 1 | 2 | 3 | 4 | 5 | *P for trend* |
| Flavonol sub-class |  |  |  |  |  |  |
| Myricetin |  |  |  |  |  |  |
| Median (mg/d) | 0.5 | 0.8 | 1.1 | 1.6 | 2.8 |  |
| No. of cases | 156 | 156 | 161 | 126 | 124 |  |
| Pooled HR (95% CI) | 1.00 (ref) | 0.93 (0.74, 1.17) | 1.00 (0.79, 1.26) | 0.81 (0.63, 1.04) | 0.87 (0.68, 1.12) | 0.21 |
| Kaempferol |  |  |  |  |  |  |
| Median (mg/d) | 0.8 | 1.4 | 2.2 | 3.6 | 7.3 |  |
| No. of cases | 155 | 158 | 154 | 135 | 121 |  |
| Pooled HR (95% CI) | 1.00 (ref) | 1.00 (0.80, 1.25) | 1.00 (0.80, 1.26) | 0.91 (0.72, 1.16) | 0.84 (0.66, 1.07) | 0.09 |
| Quercetin |  |  |  |  |  |  |
| Median (mg/d) | 5.3 | 7.9 | 10.3 | 13.4 | 20.0 |  |
| No. of cases | 150 | 147 | 141 | 161 | 124 |  |
| Pooled HR (95% CI) | 1.00 (ref) | 0.89 (0.71, 1.12) | 0.85 (0.67, 1.07) | 0.97 (0.77, 1.23) | 0.80 (0.63, 1.03) | 0.19 |
| Isohamnetin |  |  |  |  |  |  |
| Median (mg/d) | 0.0 | 0.1 | 0.3 | 0.6 | 1.4 |  |
| No. of cases | 150.0 | 152 | 145 | 137 | 139 |  |
| Pooled HR (95% CI) | 1.00 (ref) | 0.94 (0.73, 1.22) | 0.87 (0.65, 1.15) | 0.97 (0.72, 1.30) | 0.98 (0.73, 1.31) | 0.67 |
|  |  |  |  |  |  |  |
| Flavanone sub-class |  |  |  |  |  |  |
| Eriodictyol |  |  |  |  |  |  |
| Median (mg/d) | 0.01 | 0.01 | 0.02 | 0.06 | 0.21 |  |
| No. of cases | 10 | 312 | 194 | 109 | 98 |  |
| Pooled HR (95% CI) | 1.00 (ref) | 1.52 (0.79, 2.94) | 1.79 (0.94, 3.44) | 1.37 (0.71, 2.65) | 1.54 (0.79, 2.99) | 0.76 |
| Hesperetin |  |  |  |  |  |  |
| Median (mg/d) | 4.1 | 10.7 | 19.1 | 31.2 | 54.9 |  |
| No. of cases | 145 | 124 | 135 | 167 | 152 |  |
| Pooled HR (95% CI) | 1.00 (ref) | 0.84 (0.66, 1.07) | 0.86 (0.67, 1.09) | 0.96 (0.77, 1.21) | 0.79 (0.62, 1.00) | 0.18 |
| Naringenin |  |  |  |  |  |  |
| Median (mg/d) | 2.4 | 5.7 | 9.2 | 14.0 | 25.7 |  |
| No. of cases | 123 | 112 | 152 | 168 | 168 |  |
| Pooled HR (95% CI) | 1.00 (ref) | 0.81 (0.62, 1.05) | 0.96 (0.75, 1.22) | 0.95 (0.75, 1.20) | 0.83 (0.65, 1.05) | 0.26 |
| ^1^ Stratified by age, calendar time and cohort, and adjusted for menopausal status, duration of oral contraceptive use, parity, history of tubal ligation, history of hysterectomy, duration of postmenopausal hormone use by type, family history of breast or ovarian cancer, quintiles of cumulative updated energy-adjusted lactose intake and caffeine intake and quintiles of cumulative updated total energy intake. Median values in each quintile were used to test for a linear trend. Cox proportional hazards models were used for all analyses. | | | | | | |
| ^2^ Quintiles based on the distribution from pooled study population. | | |  |  |  |  |
